# “Cementing” marriages through childbearing in subsequent unions: Insights into fertility differentials among first-time married and remarried women in Ghana

**DOI:** 10.1371/journal.pone.0222994

**Published:** 2019-10-10

**Authors:** Gertrude E. Elleamoh, Fidelia A. A. Dake

**Affiliations:** Regional Institute for Population Studies, University of Ghana, Legon, Accra, Ghana; University of Botswana, BOTSWANA

## Abstract

Fertility in Ghana has declined steadily since 1980, however, a slight increase was observed between 2008 and 2014. While several factors may account for this pattern, research on the contribution of type of union is limited. This study examined differentials in the fertility of women in different types of union. Secondary data from 6,285 (weighted) ever-married women aged 15–49 years were analysed using compare means, t-test, analysis of variance, Poisson and binary logistic regression analyses. The findings indicate that, independent of other factors, fertility among remarried women is higher compared to first-time married women but this does not hold true when other factors are controlled for. Additionally, there was no significant difference in the fertility of remarried women who were in union and women who were in union in a first-time marriage. However, compared to remarried women who were currently in a union, fertility was significantly lower among remarried women who were not currently in union (β = -0.121, p<0.01) and women who have been married only once but were not currently in union (β = -0.212, p<0.001). Further analysis revealed that remarried women were significantly more likely to desire more children and less likely to use any method of contraception compared to first-time married women. There is the need for further research to better understand the fertility needs of remarried women.

## Introduction

Marriage and childbearing are integral components of cultural and social processes in Africa and because of the inter-relationship between marriage and reproduction, marriage is considered an integral part of demographic processes [1]. Furthermore, the social and legal arrangement of marriage in the sub-Saharan African region gives a couple the right to form a family as marriage is recognised as the beginning of sexual exposure, leading to childbearing [2–5]. Additionally, even though marriage and its basic function has evolved, it is still recognized as vital, because in most societies, childbearing in marriage is acceptable and a high number of births still occur within marriage [6,7].

In the sub-Saharan Africa region, even though traditional marriage and the associated cultural expectations still exist, changes in marriage and fertility patterns have been observed over the last five decades [8–11]. In recent years, some sub-Saharan African countries have experienced a stall rather than a decline in fertility and some researchers argue that, high levels of divorce and remarriage are unidentified barriers to fertility decline in the region [12]. This is an important gap in research as high rates of divorce and remarriage have previously been reported among women in some African countries. For example, divorce is reported to be relatively common in Malawi [13] and Ghana [14]. Additionally, in a study involving twenty countries in sub-Saharan Africa, Clark and Brauner-Otto (2015)[14] found that there was a linear rise in divorce as the number of years since first union among Ghanaian women increased. Furthermore, divorce has been found to be higher among women of matrilineal descent [14] and the risk of divorce is reported to be about 90% higher for women of matrilineal descent [15]. Similarly, divorce and widowhood are common in Senegal and women who are divorced or widowed often remarry and quickly, with a median duration of remarriage of one and two years respectively for widowed and divorced women [16].

In Ghana, a slight increase in total fertility rate (TFR) was observed between 2008 and 2014 following a decline between 2003 and 2008. Specifically, TFR declined from 4.3 children per woman in 2003 to 4.0 in 2008 but increased to 4.2 in 2013 and declined again to 3.9 in 2017. While there is ongoing research into possible explanations for the observed pattern of fertility in Ghana, there has been very limited research on the potential influence of type of union, particularly among women. Previous research has focused on the dichotomy of marital versus non-marital fertility or fertility differentials in polygamous versus monogamous types of unions but not much on first-time marriage versus remarriage [17]. But in a socio-cultural context where societal expectations about childbearing are high [18], such research is necessary and important as the type of union; whether first-time marriage or remarriage has implications for fertility, given that children are expected in every marital union [19,20].

More importantly, childbearing in a new union is crucial in the socio-cultural context in sub-Saharan Africa because of the unique value of children in a new union. Consequently, expectations about childbearing are high because childbearing is considered a part of the traditional marriage process and also because of the contribution of children to the family lineage [12]. Such expectations are so pervasive that soon after marriage, society expects evidence of fertility and this legitimises the marriage and until that is fulfilled, the marriage is not considered “concrete” or “cemented” [2,3,21]. In such a social milieu where a woman is expected to validate her marriage by proving her fertility in all the unions she may be involved in, fertility differentials among women who are in a first-time marriage and those who have remarried may arise. Additionally, the need to have children in subsequent unions influences certain reproductive behaviours which may impact fertility. For example, an older woman who enters a second or higher order union irrespective of the number of children she already has may not use contraception because of the desire of having children in the new union.

Against the foregoing, this study examines differentials in fertility among women in the reproductive age (15 to 49 years) from the perspective of the type of union these women are in or have been in.

## Materials and Methods

### Source of data

This study uses secondary data from the 2014 Ghana Demographic and Health Survey (2014 GDHS). The Demographic and Health Surveys (DHS) are nationally representative surveys that provide key demographic and health measures on a number of development indicators including fertility and under-five mortality, breastfeeding practices, maternal and child health, domestic violence, female autonomy, and awareness and use of family planning methods among others for the purposes of monitoring and evaluation, policy formulation and national comparability. This study used data from the women’s data file as this contains information on the variables of interest including the number of children ever born, births in the last three years, marital status, contraceptive use and other information relating to marriage and childbearing as well as socio-demographic characteristics of women in the reproductive age (15–49 years).

### Sample design and selection

The sampling frame for the 2014 GDHS was obtained from the complete list of census enumeration areas created for the 2010 Ghana Population and Housing Census. The sampling frame provided information on the location of enumeration areas, type of place of residence (rural/urban) and an estimated number of residential households. Respondents for the survey were selected through a two-stage stratified sampling procedure which made provision for all the ten regions in Ghana to be stratified by urban and rural areas using probability proportional to size of the enumeration area. At the first stage of sampling, a total of 216 and 211 enumeration areas were selected from the urban and rural strata respectively, making a total of 427 enumeration areas. In the second stage of sampling, 30 households per enumeration area were systematically selected resulting in a total of 12,831 selected households. Females aged 15–49 years in the selected households were eligible to be interviewed for the survey.

### Study Subjects

A total of 9,396 women were successfully interviewed for the 2014 GDHS. For the purpose of this study, all women between ages 15 to 49 years who have ever been married or lived with a man as if married (cohabited) were included in the analyses while those who have never been married were excluded. The sample of ever-married women also includes women who were either currently married or those who were separated, divorced or widowed at the time of the survey. The inclusion criterion of ever been married allows for women who were not married or cohabiting at the time of the survey (i.e. currently in union) but may have been married or cohabited once or more than once in the past to be included in the analysis. For those women who were currently married (at the time of the survey), they may be in a first or subsequent union. The inclusion criteria of ever been married thus allows these women to be included in the sample as well. Based on the inclusion and exclusion criterion, women who have never been married or cohabited but were within the 15–49 years age bracket were excluded from this sample because they do not have the characteristics of interest, which is, being married or in union once or more than once. An analytical sample of 6,285 (weighted) ever married women was realised after applying the inclusion and exclusion criteria and excluding missing cases (n = 13) on some variables.

### Variables

The main dependent variable for this study is the total number of children ever born which was used as an indicator of fertility and treated as a count variable. The measure comprises of all children born alive to individual women in the study sample. It is worth mentioning that number of children ever born as measured in the survey gives the number of children the women have had as at the time of the survey and thus does not necessarily indicate lifetime fertility. The main independent variable for this study is type of union, which was measured as a dichotomous variable with two categories; ‘first-time marriage’ or ‘remarriage’. The first-time marriage category denotes women who have been married or lived with a man as if married (in union) only once whereas ‘remarriage’ denotes women who have been in union more than once regardless of the number of subsequent unions they have been in. The study also controls for other proximate determinants of fertility. The proximate determinants of fertility include marital status measured as a dichotomous variable with two broad categories of ‘formerly in union’ and ‘currently in union’ and age at first cohabitation as a categorical variable with four categories of <20 years, 20–24 years, 25–29 years and 30+ years. Another variable, which is a composite variable of type of union and current marital status (interaction term) was created to further distinguish the women by the type of union they were in and their current marital status. The composite variable included four categories of; (1) married only once and currently in union, (2) married only once but not currently in union, (3) married more than once and currently in union and (4) married more than once but not currently in union. Other socio-demographic variables including age in five year age groups (15–19, 20–24, 25–29, 30–34, 35–39, 40–44 and 45–49 years), level of educational attainment (no formal education, primary, junior high, senior high and higher), religious affiliation (Catholic, Protestants, Charismatics, Other Christian, Islam and Other), lineage (matrilineal or patrilineal), type of occupation (not working, professional/technical/managerial/clerical, sales/services, agricultural and manual), place of residence (rural or urban) and wealth quintile (poorest, poorer, middle, richer and richest) were also controlled for.

### Data analysis

The characteristics of the study sample were described using means and percentages. The association between number of children ever born and the women’s socio-demographic characteristics were tested using compare means, t-test and one-way analysis of variance. Additionally, multivariate regression analysis was performed to examine the factors that influence fertility. A Poisson regression model was specified with number of children ever born which was treated as a count variable being the dependent variable. In performing the multivariate analyses, three models were specified. In the first model (Model 1), the main independent variable; type of union was regressed on the dependent variable (number of children ever born) to examine the independent effect of type of union on fertility. The second model (Model 2) controlled for the sociodemographic characteristics of the women in addition to the type of union variable. The third model further controlled for marital status in addition to the socio-demographic characteristics in Model 2. The Poisson regression analysis was also performed in a fourth model (Model 4) using the composite type of union and marital status variable as the independent variable and controlling for the other socio-demographic variables. Additional multivariate regression analysis were performed using births in the last three years, desire for more children and current contraceptive use as dependent variables to explore differences in fertility behaviours among first-time married and remarried. Poisson regression analysis was specified for the model that used births in the last three years as the dependent variable while a binary logistic regression model was specified for desire for more children (coded as no = 0 and yes = 1) and current contraceptive use (code as no method = 0 and any method = 1). The various analyses were performed in Stata version 14 and statistical significance was set at the 5% alpha level (p<0.05).

## Results

### Characteristics of the study sample

The results in Table 1 show that the average number of children ever born to the women in the study was approximately 3.4. In terms of the type of union the women were in, about 23 percent have been in union more than once and about two-thirds (65.7%) were currently in a first union (at the time of the survey) while a little less than one fifth (18.8%) were in a second or higher order union. Also, a little over one-tenth (11.6%) of the women have been married once but were not currently in union and about 4% have been married more than once but were not in union at the time of the survey (Table 1). More than half (53.8%) of the women first cohabited before age 20 and in terms of current age, about one-fifth of the women were aged 30–34 and 35–39 years. Regarding education, about a quarter of the women had no formal education and only a small proportion had higher than secondary level of education (Table 1). Over 70 percent of the women were of different Christian faiths and in terms of lineage, a little over half (50.8%) were of patrilineal descent. The distribution by type of occupation shows women who were engaged in professional/technical/managerial/clerical types of work being in the minority and in terms of place of residence, a little over half of the women lived in urban areas. Regarding wealth status, those belonging to the richest quintile formed the highest proportion (Table 1).

**Table 1:** Background characteristics of women.

Variable	Mean/Percentage
**Number of children ever born (Mean ± SD)**	3.39 ± 2.22
**Type of union**	** **
First-time marriage	77.2
Remarriage	22.8
**Marital status**	
Formerly in union	15.6
Currently in union	84.4
**Composite type of union and marital status**	
Married only once and currently in union	65.6
Married only once but not currently in union	11.6
Married more than once and currently in union	18.8
Married more than once but not currently in union	4.0
**Age at first cohabitation**	
< 20	53.8
20–24	29.1
25–29	12.8
30+	4.3
**Current Age**	** **
15–19	1.9
20–24	10.8
25–29	18.6
30–34	19.6
35–39	19.6
40–44	16.1
45–49	13.5
**Level of education**	
No education	26.3
Primary	19.2
Junior high	40.1
Senior high	9.4
Higher	4.9
**Religion**	
Catholic	9.8
Protestants	12.7
Charismatic	41.1
Other Christian	14.9
Islam	15.5
Other	5.9
**Lineage**	
Matrilineal	49.2
Patrilineal	50.8
**Occupation**	
Not working	12.2
Prof/Tech/clerical	5.7
Sales/Services	44.5
Agricultural	24.4
Manual Labour	13.2
**Type of place of residence** Urban	51.1
Rural	48.9
**Wealth index**	** **
Poorest	17.6
Poorer	18.1
Middle	20.6
Richer	21.3
Richest	22.4
**Total % (N)**	**100 (6285)**

Source: Generated from GDHS, 2014

### Variations in number of children ever born

The variations in the number of children ever born to the women by their socio-demographic characteristics are shown in Table 2. The results indicate that women who were remarried have at least one child more than women who have been married only once (4.18 and 3.15 respectively). Similarly, those who were in union at the time of the survey had 0.25 more children than women who were formerly in union. Considering the composite of type of union and marital status, the results reveal that fertility is highest among women who are currently in a remarriage (4.21) and lowest among those who have been married only once but were not currently in a union (2.90). Considering the other variables included in the analysis, the results generally show that women who first cohabited at younger ages had more children than women who first cohabited at older ages and fertility tends to increase with age, with women in the oldest age-group having the highest number of children ever born. Considering education, it was observed that every higher level of education attained is accompanied by a further decline in the mean number of children ever born. In terms of religious affiliation, the fertility of Christians was collectively lower than that of Muslims and Other religious groups and in terms of occupation, women in professional/technical/managerial/clerical occupations had less children compared to those who were not working. The results also reveal that women residing in rural areas had higher fertility; about one more child than their counterparts in urban areas. And the results with regards to wealth status indicate that fertility declined with increasing wealth quintile (Table 2).

**Table 2:** Mean number of children ever born by background characteristics of women.

Variable	Mean (SD)	F (p-value)
**Type of union**	** **	**244.72 (0.000)**
First-time marriage	3.15 (2.14)	
Remarriage	4.18 (2.31)	
**Marital status**	** **	**10.08 (0.002)**
Formerly in union	3.18 (2.08)	
Currently in union	3.43 (2.25)	
**Composite of type of union and marital status**		**86.34 (0.000)**
Married only once and currently in union	3.20 (2.17)	
Married only once but not currently in union	2.90 (1.95)	
Married more than once and currently in union	4.21 (2.32)	
Married more than once but not currently in union	3.99 (2.23)	
**Age at first cohabitation**	** **	**161.99 (0.000)**
< 20	3.89 (2.29)	
20–24	3.08 (2.03)	
25–29	2.40 (1.80)	
30+	2.14 (1.89)	
**Current age**	** **	**459.28 (0.000)**
15–19	0.88 (0.68)	
20–24	1.49 (1.01)	
25–29	2.14 (1.28)	
30–34	3.13 (1.73)	
35–39	3.88 (1.94)	
40–44	4.73 (2.30)	
45–49	5.02 (2.51)	
**Educational level**	** **	**269.95 (0.000)**
No education	4.47 (2.41)	
Primary	3.78 (2.32)	
Junior High	3.04 (1.86)	
Senior High	1.94 (1.40)	
Higher	1.71 (1.41)	
**Religion**	** **	**30.90 (0.000)**
Catholic	3.37 (2.18)	
Protestant	3.07 (2.14)	
Charismatic	3.21 (2.08)	
Other Christian	3.35 (2.27)	
Islam	3.72 (2.34)	
Other	4.52 (2.54)	
**Lineage**	** **	**3.36 (0.067)**
Matrilineal	3.34 (2.20)	
Patrilineal	3.44 (2.25)	
**Occupation**	** **	**215.91 (0.000)**
Not working	2.74 (2.00)	
Pro/tech/clerical	1.96 (1.52)	
Sales/servicing	3.14 (2.00)	
Agricultural	4.65 (2.41)	
Manual labour	3.11 (2.02)	
**Type of place of residence**	** **	**320.72 (0.000)**
Urban	2.91 (2.00)	
Rural	3.89 (2.34)	
**Wealth index**	** **	**218.91 (0.000)**
Poorest	4.38 (2.52)	
Poorer	4.24 (2.33)	
Middle	3.44 (2.11)	
Richer	2.87 (1.84)	
Richest	2.36 (1.65)	
**Total Mean**	**3.39 (2.22)**	** **

SD = Standard Deviation

Source: Generated from GDHS, 2014

### Predictors of children ever born and other fertility indicators/behaviours

The results of the unadjusted model (Model 1, Table 3) with type of union as the main predictor variable suggest that women in remarriage have higher fertility than their counterparts in first-time marriages. However, this did not hold true when other factors were controlled for. Type of union was no longer statistically significant in predicting children ever born in the adjusted model with type of union and socio-demographic characteristics as the independent variables (Models 2), or the model that further controlled for current marital status (Model 3). Furthermore, the results of the model with the composite type of union and marital status variable as the main predictor variable and controlling for other variables show that there is no significant difference in the fertility of women who are remarried and are currently in union and women who are currently in union in a first-time marriage (Model 4). However, compared to women who were remarried and in union at the time of the survey, fertility was lower among women who have been married only once but were not currently in union (β = -0.212, p<0.001) and remarried women who were not currently in union (β = -0.121, p<0.01) (Model 4). The results for the other fertility indicators/behaviours show that fertility in the last three years is not significantly different among remarried women and first-time married women but remarried women are more likely to want to have more children and they are less likely to use any method of contraception compared to women in a first-time marriage (Table 4).

**Table 3:** Predictors of fertility by type of union.

Variable (RC)	Model 1 β (s.e.)	Model 2 β (s.e.)	Model 3 β (s.e.)	Model 4 β (s.e.)
**Type of union** (First-time marriage)				
Remarriage	0.281 (0.021)***	0.009 (0.017)	0.002 (0.017)	
**Marital status** (Formerly in union)				
Currently in union			0.194 (0.021)***	
**Composite of type of union and marital status (**Married more than once and currently in union)				
Married only once and currently in union				0.015 (0.018)
Married only once but not currently in union				-0.212(0.028)***
Married more than once but not currently in union				-0.121 (0.039)**
**Age at first cohabitation** (< 20)				
20–24		-0.205 (0.016)***	-0.212 (0.016)***	-0.212 (0.016)***
25–29		-0.369 (0.030)***	-0.376 (0.029)***	-0.377 (0.029)***
30+		-0.644 (0.060)***	-0.651 (0.058)***	-0.651 (0.058)***
**Current age (**15–19)				
20–24		0.617 (0.080)***	0.620 (0.079)***	0.620 (0.079)***
25–29		1.102 (0.078)***	1.103 (0.077)***	1.104 (0.077)***
30–34		1.512 (0.078)***	1.521 (0.077)***	1.521 (0.077)***
35–39		1.723 (0.078)***	1.739 (0.077)***	1.739 (0.077)***
40–44		1.858 (0.078)***	1.876 (0.077)***	1.879 (0.077)***
45–49		1.883 (0.078)***	1.918 (0.077)***	1.919 (0.077)***
**Educational level** (No education)				
Primary		-0.033 (0.020)	-0.026 (0.020)	-0.027 (0.020)
Junior High		-0.112 (0.021)***	-0.104 (0.020)***	-0.103 (0.020)***
Senior High		-0.290 (0.037)***	-0.280 (0.037)***	-0.279 (0.037)***
Higher		-0.342 (0.070)***	-0.336 (0.070)***	-0.336 (0.070)***
**Religion (**Catholic)				
Protestants		-0.001 (0.031)	0.002 (0.030)	0.004 (0.030)
Charismatic		0.016 (0.022)	0.016 (0.022)	0.017 (0.022)
Other Christian		0.034 (0.026)	0.031 (0.026)	0.031 (0.026)
Islam		0.076 (0.024)**	0.060 (0.023)*	0.059 (0.023)*
Other		0.078 (0.030)**	0.080 (0.030)**	0.081 (0.030)**
**Lineage** (Patrilineal)				
Matrilineal		0.094 (0.016)***	0.097 (0.016)***	0.097 (0.016)***
**Occupation** (Not working)				
Prof/Tech/Clerical		-0.128 (0.053)*	-0.132 (0.054)*	-0.131 (0.054)*
Sales/Services		-0.089 (0.026)**	-0.084 (0.026)**	-0.083 (0.026)**
Agricultural		0.012 (0.027)	0.004 (0.027)	0.005 (0.027)
Manual		-0.109 (0.031)***	-0.102 (0.030)***	-0.102 (0.030)***
**Type of place of residence** (Urban)				
Rural		0.001 (0.019)	-0.007 (0.019)	-0.008 (0.019)
**Wealth Index** (Poorest)				
Poorer		-0.065 (0.019)**	-0.056 (0.019)**	-0.057 (0.019)**
Middle		-0.178 (0.023)***	-0.161 (0.023)***	-0.162 (0.023)***
Richer		-0.266 (0.029)***	-0.260 (0.028)***	-0.259 (0.028)***
Richest		-0.349 (0.035)***	-0.361 (0.034)***	-0.362 (0.034)***
Constant	1.149 (0.012)*	0.027 (0.081)	-0.153 (0.082)	-0.091 (0.089)
Wald chi^2^	(1) 178.43	(29) 5190.11	(30) 5438.42	(31) 5490.02
Prob > chi^2^	0.0000	0.0000	0.0000	0.0000
Pseudo R^2^	0.0118	0.1821	0.1856	0.1858
Log pseudolikelihood	-13593.931	-11251.609	-11203.018	-11199.858

RC = Reference Category s.e. = standard error

*p < 0.05

**p < 0.01

*** p < 0.001

Source: Computed from GDHS, 2014

**Table 4:** Multivariate regression results showing predictors of births in the last three years, desire for more children and current contraceptive use.

	Dependent variables		
Independent variables ^(RC)^	Births in the last three years β (s.e.)	Desire more children Odds ratio	Current Contraceptive Use Odds ratio
**Type of union** ^(First-time marriage)^			
Remarriage	0.013 (0.045)	1.258*	0.745**
**Marital status** ^(Formerly in union)^			
Currently in union	0.786 (0.094)***	0.723**	1.380**
**Age at first cohabitation** ^(< 20)^			
20–24	0.140 (0.039)***	1.620***	0.857
25–29	0.240 (0.063)***	2.610***	0.793
30+	0.448 (0.094)***	5.610***	0.341***
**Current age** ^(15–19)^			
20–24	0.033 (0.091)	0.450	2.152*
25–29	0.016 (0.093)	0.173***	2.175**
30–34	-0.060 (0.094)	0.058***	1.857*
35–39	-0.427 (0.101)***	0.025***	1.851*
40–44	-1.098 (0.114)***	0.010***	1.686
45–49	-2.293 (0.159)***	0.006***	1.016
**Educational level** ^(No education)^			
Primary	-0.001 (0.048)	0.735*	1.571***
Junior High	-0.064 (0.046)	0.821	1.490***
Senior High	-0.209 (0.077)**	1.016	1.906***
Higher	-0.068 (0.135)	0.316***	2.243**
**Religion** ^(Catholic)^			
Protestants	0.103 (0.071)	0.937	0.979
Charismatic	0.065 (0.204)	0.970	1.039
Other Christian	0.092 (0.065)	0.675*	1.237
Islam	0.167 (0.058)**	2.207***	0.691
Other	0.295 (0.068)***	1.385	0.822
**Lineage** ^(Patrilineal)^			
Matrilineal	0.102 (0.042)*	0.807*	1.087
**Occupation** ^(Not working)^			
Prof/Tech/Clerical	-0.438 (0.121)***	3.216***	1.217
Sales/Services	-0.316 (0.053)***	1.432*	1.309*
Agricultural	-0.201 (0.057)***	1.318	1.266
Manual	-0.345 (0.064)***	1.373	1.258
**Type of place of residence** ^(Urban)^			
Rural	-0.013 (0.045)	1.149	1.308*
**Wealth Index** ^(Poorest)^			
Poorer	-0.006 (0.047)	0.560***	1.195
Middle	-0.261 (0.057)***	0.768	1.097
Richer	-0.235 (0.064)***	0.927	1.163
Richest	-0.370 (0.081)***	1.100	1.116
**Constant**	-0.795 (0.141)***	29.365***	0.075***

RC = Reference Category s.e. = standard error

*p < 0.05

**p < 0.01

*** p < 0.001

Source: Generated from GDHS, 2014

## Discussion

This study sought to examine differentials in fertility based on type of union (whether first-time or subsequent union) among women in the reproductive age in Ghana. The findings indicate that the number of unions a woman has been is only significantly associated with fertility in the absence of other factors but when other factors are considered there is no significant difference in the fertility of first-married women and remarried women Additionally, the findings from modelling the interaction between type of union and current marital status and controlling for other factors show that fertility among women who have been married more than once and are in union is not different from fertility among first-time married women who are in union. However, fertility among remarried and first-time married women who are not in union is significantly lower than among remarried women who are in union.

The findings showing fertility being lower among remarried women who are not currently in union compared to remarried women who are in union are plausible because of the socio-cultural expectation of female reproduction that structures the life course of an African woman around marriage, child bearing and raising children [18,22]. As such, if a woman experiences any marital disruption, she is often expected to quickly remarry in order to get back on her life’s course [3,18,22] and resume the role of reproduction. Additionally, the societal expectation of women proving their fertility evidenced by the manifestation of a birth and the use of children as a means of stabilising unions [18,23–26] makes having children in remarriages all the more important. It is the expectation that the seal of a long-lasting partnership between married couples, which is a child(children) should be present in each subsequent union regardless of the number of children couples may have had before forming a new union and this is because of the unique position children hold in unions [12]. Additionally, the findings of the composite type of union and marital status further emphasize the need to have children not only because of being remarried but also because of being in a subsequent union. Against the foregoing, it is expected of a woman who has been in subsequent unions to have more children compared to a woman who apart from the first marriage has not been in any other union. Again, considering the other dependent variables that were examined in this study to explore the possible drivers of fertility differentials among remarried and first-time married women, it was found that remarried women were more likely to desire more children. Remarried women were also less likely to use any method of contraception and this could probably be because the need to have children may be higher for remarried women than women who are in a first-time union.

With regards to other factors that potentially account for the differentials in fertility, it was found that age at first cohabitation, current age, level of educational attainment, lineage and wealth status significantly predict fertility. The inverse relationship between education and fertility as was found in the current study is consistent with findings from other similar studies [27–34]. Furthermore, it was found that fertility was higher among women of matrilineal descent. This could be because of the higher risk of divorce among matrilineal women [14,15] which potentially influences the need to have more children in a subsequent union. Another finding in the present study that is worth mentioning is the fertility differentials by location of residence. While several studies have found higher levels of fertility in rural compared to urban areas [20,31,35–39], in the present study, there was no significant difference in the fertility of women who reside in rural areas and those who reside in urban areas.

Summing up, the findings of this study make useful contributions to fertility research in Ghana and other African countries with similar socio-cultural context where marriage and fertility form an integral part of the social structure of society and institutions. The findings indicate that there is the need to understand the fertility needs of couples, particularly women in the context of different types of union be it a first-time or subsequent union. The study findings are generalizable to Ghanaian women between the ages of 15 to 49 years as the study uses nationally representative data from the recent round of the demographic and health survey and makes the necessary analytical adjustments using the appropriate sample weighting procedures. However, as the study uses secondary data, the findings are not without some limitations. Firstly, the number of children ever born includes the total number of children born to a woman and this was not disaggregated into those children she had in either a first-time marriage or higher order marriage or in each union. It was thus not possible to disaggregate the number of children born to a woman by the number of unions she has been in. For instance, a woman who has been in more than one union and had all her children in the first union, would be classified under remarriage with the children who should have been classified under the first-time marriage but because this disaggregation was not available, this distinction could not be made. Secondly, the time lapse between unions especially among remarried women is not accounted for in the data. Controlling for the time lapse between unions may impact the results differently but this could not be investigated in the current study because there is no measure of this in the data. Thirdly, it was not possible to investigate whether women who remarried did so because of they became pregnant or that women who may not have had children in a previous union got divorced and remarried in order to have children in a subsequent union. Fourthly, the data does not give explicit information on lineage. Therefore using ethnicity as a proxy for lineage and categorising the various ethnic groups as Akan (matrilineal) and non-Akan (patrilineal) as was done in the current study may not give a correct measure of lineage as some Akans are bilateral rather than belonging to one distinct lineage, be it matrilineal or patrilineal. These limitations notwithstanding, the findings of this study are valid but should be interpreted taking into consideration the possible effects of the limitations cited.

## Conclusion

The findings of this study indicate that there is no significant difference in the fertility of first-time married and remarried women between the ages of 15 to 49 years in Ghana when other factors are considered. However, fertility is lower among women who have been in union only once but are not currently in union and remarried women who are not currently in union. Thus being in union in a subsequent union is associated with higher fertility. The desire of remarried women to have more children coupled with their lower likelihood of using contraception may be driving this finding and this may have implications for reducing fertility. There is the need for further research to understand the dynamics of fertility among women who are remarried or in subsequent unions and how a reduction in their fertility, if at all can be achieved.
